# Bayerisches Meldeportal für Corona-Reihenuntersuchungen – Entwicklung und Einsatz einer digitalen Lösung in der Pandemie

**DOI:** 10.1007/s00103-022-03639-3

**Published:** 2023-01-17

**Authors:** Jessica Flöter, Julia Roll, Anna Kirchner, Chu-Wei Hung, Anna Riedl, Katharina Kotula, Ursula Mühle-Schäffer, Johanna Tomandl, Thomas Ewert, Ralf Heinrich, Christoph Schlegel, Christian Markl, Uta Nennstiel, Bernhard Liebl, Christian Weidner, Manfred Wildner, Thomas Keil, Carolin Stupp

**Affiliations:** 1 Landesinstitut für Gesundheit I, Bayerisches Landesamt für Gesundheit und Lebensmittelsicherheit, Erlangen, Deutschland; 2 Landesinstitut Gesundheit II, Bayerisches Landesamt für Gesundheit und Lebensmittelsicherheit, Erlangen, Deutschland; 3 Landesinstitut Bayerisches Krebsregister, Bayerisches Landesamt für Gesundheit und Lebensmittelsicherheit, Erlangen, Deutschland; 4 Institut für klinische Epidemiologie und Biometrie, Universität Würzburg, Würzburg, Deutschland; 5 IT-Dienstleistungszentrum des Freistaats Bayern, Landesamt für Digitalisierung, Breitband und Vermessung, Marktredwitz, Deutschland; 6 Walther-Straub-Institut für Pharmakologie und Toxikologie, Ludwig-Maximilians-Universität München, München, Deutschland; 7 Bayerisches Landesamt für Gesundheit und Lebensmittelsicherheit, Erlangen, Deutschland; 8 Pettenkofer School of Public Health, Ludwig-Maximilians-Universität München, München, Deutschland; 9 Institut für Sozialmedizin, Epidemiologie und Gesundheitsökonomie, Charité – Universitätsmedizin Berlin, Berlin, Deutschland

**Keywords:** Meldesoftware, Digitalisierung, SARS-CoV‑2, Tests, Gesundheitsamt, Software for monitoring, Digitalization, SARS-CoV‑2, Screening tests, Health authorities

## Abstract

Zur Unterstützung der Entscheidungsträger werden in der SARS-CoV-2-Pandemie unterschiedlichste Daten erhoben, um ein möglichst genaues Lagebild zur Steuerung von Pandemiemaßnahmen zu erhalten. Nur wenig hilfreich sind dabei sowohl Papier und Stift als auch der Versand einzelner medienbruchbehafteter Dateien, die später zusammengefügt werden müssen. Es wird eine solide und zeitnah zur Verfügung stehende Datengrundlage benötigt. Das Bayerische Meldeportal für Corona-Reihenuntersuchungen (BayCoRei) stellt mit der gewählten Systemarchitektur eine zentrale bayernweit einheitliche digitale Lösung dar, die agil und einfach zu bedienen ist. BayCoRei greift auf bereits etablierte technische Komponenten und Schnittstellen zurück. Neben den technischen Komponenten spielt die Unterstützung der einzelnen Akteure (Gesundheitsämter, Leistungserbringer, Bezirksregierungen etc.) eine entscheidende Rolle für den Erfolg der Lösung. Dieser Beitrag zeigt am Beispiel von BayCoRei und 2 anderen Online-Meldeportalen, welche Technik und Architektur sich für einen (eiligen) Aufbau etabliert haben, aber ebenso, wo sich Wunsch und Wirklichkeit auch im Hinblick auf die Steuerung der Pandemiemaßnahmen unterscheiden.

## Abstract (en)

During the SARS-CoV‑2 pandemic, various data had to be collected to support political decisions for pandemic preparedness and response. Nevertheless, using analogue tools like paper and pencil as well as sending files with media discontinuity that have to be merged later are not useful and can hardly provide usable data in real time. With the selected system architecture, the Bavarian Online Database for Corona Screening Tests (BayCoRei) is a central, Bavaria-wide, consistent digital solution that is agile and easy to use. BayCoRei uses established technical components and interfaces. Apart from this, the support of the individual stakeholders (e.g., health authorities, service providers, and district governments) plays a decisive role in the success of the solution. The present article describes BayCoRei and two other online databases as examples that comprise the technology and architecture that have proven to be (rapidly) deployable and points out the gap between intention and reality regarding pandemic management.

## Einleitung

„We have a simple message for all countries: Test, test, test. Test every suspected case“ [1]. So appellierte der Generaldirektor der Weltgesundheitsorganisation (WHO) Dr. Tedros Ghebreyesus am 16.03.2020 in der damals regulär stattfindenden Pressekonferenz an die Nationen. Ghebreyesus unterstrich die Bedeutung der Testung von Verdachtsfällen für die Unterbrechung von Infektionsketten als entscheidenden Baustein zur (SARS-CoV-2-)Pandemiebekämpfung.

Brauchbare Daten, bestenfalls in Echtzeit, sind nicht erst in der Pandemie zu einer möglichen wertvollen Ressource als Steuerfaktor für Wissenschaft und Politik geworden, aber in einer „gesundheitlichen Notlage von internationaler Tragweite“ (GNIT) nach Artikel 12 der internationalen Gesundheitsvorschriften (IGV) der WHO besonders notwendig. In einem volatilen und damit auch unübersichtlichen Geschehen wurden zu Beginn der Pandemie verschiedene Daten gesammelt, um sich ein möglichst übersichtliches Lagebild als Entscheidungsgrundlage zu verschaffen. Dabei kam Testungen auf Vorliegen einer SARS-CoV-2-Infektion eine besondere Bedeutung zu.

In der frühen Phase der Pandemie hatte die Bayerische Staatsregierung in der Kabinettssitzung am 30.06.2020 beschlossen, vorrangig Verdachtsfälle zu testen, gleichzeitig aber auch ihre Teststrategie zur Bewältigung der Corona-Pandemie systematisch zu differenzieren und auszuweiten [2]. Ab diesem Zeitpunkt wurden sowohl gezielte Reihenuntersuchungen, insbesondere in Einrichtungen mit vulnerablen Personengruppen durchgeführt als auch Testungen für jedermann ermöglicht.

Die Leistungserbringer (Ärzte oder Gesundheitspersonal und im Pandemieverlauf auch andere fachkundig geschulte Personen, die Tests durchführen) wurden, neben den Meldepflichten nach *Infektionsschutzgesetz *(IfSG)^1^, mit der *Coronavirus-Testverordnung* (TestV)^2^ und der Bayerischen Teststrategie (01.07.2020) verpflichtet, die Testdaten erbrachter, vorrangig asymptomatischer Tests über die Gesundheitsämter (GÄ) und Regierungen an das Bayerische Staatsministerium für Gesundheit und Pflege (StMGP) zu berichten. Eine Meldung der Daten bspw. über einzelne Excel-Dateien der jeweiligen Leistungserbringer wäre nicht zu bewältigen gewesen. Daher wurde zur erleichterten Erfüllung der Berichtspflichten für die GÄ sehr schnell eine Online-Plattform für die landesweit einheitliche, strukturierte Erfassung von Testergebnissen entwickelt.

Im vorliegenden Beitrag wird das Bayerische Meldeportal für Corona-Reihenuntersuchungen (BayCoRei) als Fallbeispiel für die eilige Implementierung einer digitalen Lösung während der frühen Phase der Corona-Pandemie vorgestellt. Neben einzelnen Grunddaten bzw. zu erfassenden Merkmalen und den vorgesehenen Funktionalitäten zur ersten Einordnung des Portals werden vor allem die technischen Rahmenbedingungen skizziert. Die Architektur, die Einbettung, die Einführung (Rollout), der Betrieb und die Weiterentwicklung des Portals werden beschrieben. Im Weiteren werden die Möglichkeiten erläutert, wie BayCoRei die Steuerung der Pandemiemaßnahmen unterstützen kann, und diskutiert, welche technischen Möglichkeiten das Portal bietet.

## Allgemeine Anforderungen an das Meldeportal

Aufgrund ihrer naturgemäß i. d. R. hohen Dynamik in Hinblick auf die Erfassung von Testdaten, Daten zu klinischen Verläufen oder zum Immunstatus in der Bevölkerung stellt eine Pandemie viraler Atemwegserkrankungen besondere Anforderungen an eine Software. Erforderlich ist eine schnell verfügbare bzw. realisierbare sowie im Verlauf rasch anpassungsfähige, gleichzeitig auch allseits robuste, unter Daten- und Cybersicherheitsaspekten geeignete und bestenfalls bereits etablierte Lösung. Die Anwendung sollte für die verschiedenen Nutzergruppen einfach zu bedienen und die berichteten Daten sollten zeitnah und bedarfsorientiert verfügbar sein.

**Figure 1:**
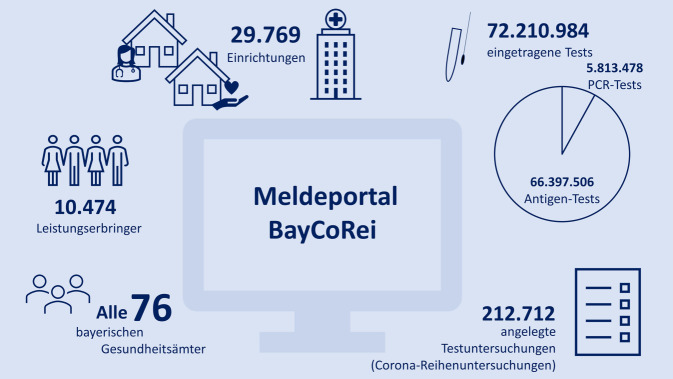

BayCoRei wurde vor diesem anspruchsvollen Anforderungsprofil im Juli/August 2020 als digitale Softwarelösung konzipiert, umgesetzt und eingeführt. Innerhalb weniger Tage war es aufgrund der ausgeweiteten bayerischen Teststrategie ad hoc erforderlich, über eine anfängliche Stichprobe von begrenzten Einrichtungszahlen und Einrichtungsarten hinaus, eine möglichst breite Datenerfassung abzubilden. Das Online-Portal sollte allen berichtspflichtigen, vorrangig medizinischen Einrichtungen die Eintragung der SARS-CoV-2-*polymerase chain reaction*(PCR)-Testergebnisse ermöglichen, aber ggf. auch die Erfassung bspw. der Schultestungen zulassen. Abb. 1 stellt einzelne zentrale Kennzahlen zu BayCoRei dar.

Grundsätzlich sollten mit dem Meldeportal größere Datenmengen von mehreren Tausend Einrichtungen einfach und wenig fehleranfällig erfasst und effektiv verwaltet werden können. Mit einer landesweit einheitlichen Lösung sollten nicht nur Risiken, die mit der Nutzung diverser Meldewege und -medien einhergehen, umgangen, sondern auch die Vorteile einer übergreifenden Datensammlung genutzt werden. Zur Entlastung der pandemiebedingt ohnehin stark beanspruchten und mit der Erfassung betrauten GÄ sollten die digitale Lösung sowie alle damit verbundenen Überlegungen und Anstrengungen zentral geleistet werden.

Die Daten der Reihenuntersuchungen (d. h. Testungen bei primär Gesunden zur Früherkennung) sind Ausgangsbasis für eine mögliche Darstellung von Häufigkeiten, regionalen Mustern und zeitlichen Verläufen von Infektionen bei asymptomatischen Mitarbeitern, Patienten, Bewohnern, Besuchern und anderen Personengruppen. Dazu werden v. a. die kumulierten Testergebnisse (Gesamtzahl der Tests, positive und/oder negative Testergebnisse) aus Reihenuntersuchungen in Kliniken und Heimen, welche bspw. bei Mitarbeitern oder Bewohnern durchgeführt werden, jeweils nach Zeiteinheiten (z. B. Woche, Monat) erfasst.

Seit dem 01.08.2021 sind die erbrachten Testungen und die Zahl der positiven Testergebnisse der Bürgertestungen nach § 4a TestV aufgrund einer Verordnung nicht wie die anderen Daten der Reihenuntersuchungen nur zu berichten, sondern verpflichtend zu melden (§ 7 Abs. 10 TestV). In Bayern wird diese Meldepflicht mit Hilfe von BayCoRei erfüllt. Gemäß den weiteren Vorgaben zur TestV wurden bzw. werden die durch die GÄ angelegten und gepflegten Einrichtungsdaten zugleich zur Datenverifizierung für die Freischaltung der Leistungserbringer für die Corona-Warn-App (CWA) sowie zur Abrechnungsprüfung für die Kassenärztliche Vereinigung Bayerns (KVB) herangezogen (§ 7a TestV). Die KVB erhält ferner die Gesamt- und Positivenzahl der durchgeführten und durch die Leistungserbringer eingetragenen Bürgertests.

Die folgenden Abschnitte geben einen Überblick, welche Merkmale erfasst werden, welche Funktionalitäten (pandemiebedingt) gefordert werden, welche technische Architektur zum Gelingen der Umsetzung beigetragen hat und wie diese strukturell und prozessual eingebettet wurde. Die Abschnitte zur Umsetzung und zum Betrieb des Meldeportals verdeutlichen, welche Agilität der technischen Lösung, aber auch den Umsetzungsbeteiligten abverlangt wurde und welchen Wandel das Portal erfuhr.

## Merkmale, die im Meldeportal erfasst werden

**Figure 2:**
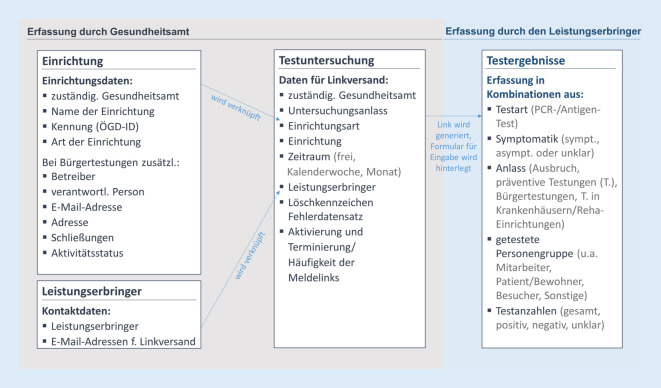

Abb. 2 zeigt in Hauptkategorien zusammengefasst, welche Merkmale aktuell zur Erfassung der Testergebnisse und der anschließenden Auswertung von regionalen Mustern und zeitlichen Verläufen erhoben werden. Die zu erfassenden Merkmale wurden bis auf wenige Streichungen fortlaufend situativ erweitert. Durch die Kombination mehrerer Dimensionen (bspw. Testart PCR und/oder Antigen (AG) und Symptomatik Ja/Nein) wird die Erfassung der Merkmale mit ihren variierenden Parametern (Merkmal: getestete Mitarbeiter, Parameter: Anlass Ausbruch, Anlass präventive Testung …) technisch in möglichst wenigen Spalten/Variablen realisiert. Die Testergebnisse werden in BayCoRei aggregiert und anonymisiert ohne Personenbezug erfasst. Zudem wurde ein Rechte- und Rollenkonzept umgesetzt, in dessen Rahmen die GÄ nur auf Daten von Einrichtungen zugreifen können, die im Zuständigkeitsbereich des jeweiligen Gesundheitsamts (GA) liegen.

## Technische Architektur und Einbettung

**Figure 3:**
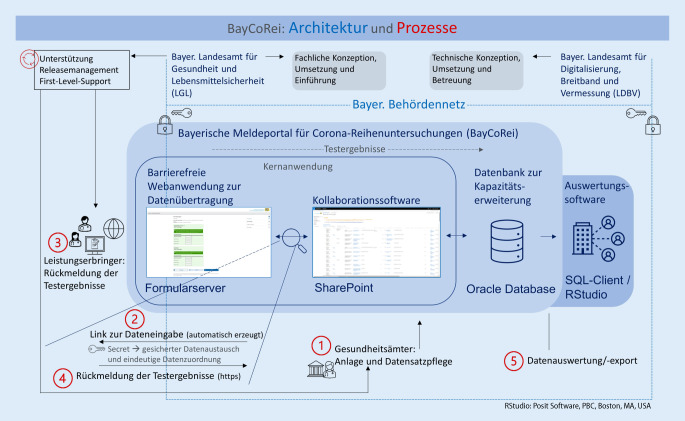

Die technische Lösung wurde auf Basis der fachlichen Konzeption des Bayerischen Landesamts für Gesundheit und Lebensmittelsicherheit (LGL) durch den eigenen zentralen IT-Dienstleister des Freistaats Bayern (IT-DLZ) am Landesamt für Digitalisierung, Breitband und Vermessung (LDBV) entwickelt und umgesetzt. Zur schnellen und tauglichen Umsetzung wurde auf folgende bereits vorhandene und etablierte technische Komponenten des staatlichen IT-DLZ zurückgegriffen, die im Sinne eines „Minimum Viable Product“^3^ zu einer Lösung (BayCoRei) verschmolzen wurden: ein barrierefreier Webassistent zur Datenübertragung (Formularserver – FS; cit intelliForm Server: cit GmBH, 73265 Dettingen/Teck, Deutschland), eine Kollaborationssoftware (SharePoint – SP; Microsoft Corporation, Redmond, WA, USA), eine zusätzliche Datenbank (Oracle – OD; Oracle Corporation, Austin, TX, USA) sowie Schnittstellen zwischen den Komponenten (Abb. 3). Vorteile dieser bereits erfolgreich etablierten Strukturen waren bspw. die geregelten Anforderungen bzgl. Datensicherheit und -schutz, Benutzerverwaltung, Zugriffsrechten, Systemstabilität sowie die regelmäßigen Routine-Updates im Rahmen der bestehenden Infrastruktur und die vorhandenen Erfahrungen sowohl beim IT-DLZ als auch beim LGL.

Alle Komponenten vereint, dass sie niedrigschwellig und ohne große Hürden schnell eingesetzt werden konnten, aber auch, wie pandemiebedingt zu erwarten, eine gewisse Offenheit hatten, agil weiterentwickelt werden zu können. Die zentralen Gründe für die Nutzung der ausgewählten Komponenten sind Tab. 1 zu entnehmen. Im Folgenden werden der Aufbau von BayCoRei und das Zusammenwirken der verschiedenen technischen Komponenten sowie deren Bedeutung bei der Meldung der Testergebnisse vertieft (Abb. 3). Die Kernanwendung von BayCoRei bildet eine einfache FS-SP-Kopplung, mit der die Testergebnisse durch die Leistungserbringer im Internet erfasst und nach der gesicherten Übertragung in den SP im bayerischen Behördennetz dort gespeichert und verwaltet werden können. Das Behördennetz ist eine abgeschlossene, gesicherte IT-Infrastruktureinheit, die der Kommunikation der Behörden und Behördendienste untereinander dient [4].

**Table 1:** 

Gründe für die Nutzung	Technische Komponenten			
	Webassistent zur Datenerfassung Formularserver	Kollaborationssoftware SharePoint	Datenbank Oracle	Nutzung Behördennetz
Etabliertes System	+	+	+	+
Schnelle Umsetzbarkeit	+	+	+	+
Anpassungsfähigkeit	+	+	+	/
Etablierte Lösung des IT-DLZ	+	+	+	+
Weitere zentrale Auswahlkriterien	Handling eines großen Nutzerkreises (mehrere 10.000) außerhalb des Behördennetzes; ohne Einsatz einer eigenen Benutzerverwaltung	Durch leichtgewichtiges, initiales Setup schnell einsetzbare Software mit Datenbank	Verarbeitung und Speicherung großer Datenmengen bei gleichzeitigem Erhalt der SharePoint-Performance	/
	Verschlüsselte Übertragung (https)	Spätere Ergänzung schwergewichtiger Programmelemente möglich	Weitere Standardauswertungen, z. B. per SQL, möglich	
		Anbindung an weitere bestehende Systeme (bei Bedarf) möglich		

*IT-DLZ* IT-Dienstleistungszentrum des Freistaates Bayern, *SQL* Structured Query Language (dt.: strukturierte Abfragesprache)

### SharePoint

Der SP ist das Herzstück von BayCoRei und kann nahezu das gesamte Meldeverfahren, inkl. Benutzerverwaltung und Datenmanagement, abbilden. Einzig die Testergebniseingabe durch Nutzer außerhalb des Behördennetzes erfolgt durch den FS und die Speicherung der inzwischen großen Datenmengen durch eine größere OD. Die aktuell beim IT-DLZ ausgestaltete SP-Version orientiert sich am Bedarf des gesamten Freistaats und liefert eine ausreichende Performance bis zu den jeweiligen zweckmäßigen Kapazitätsgrenzen. Diese sind nicht exakt zu benennen, weil sie in Abhängigkeit des Ressourcenaufwands durch ergänzende Programmierungen veränderbar sind. Zur Sicherstellung der optimalen Performance wird der SP oberhalb der vorgegebenen Kapazitätsgrenzen standardmäßig durch eine OD ergänzt, in der die ältesten Datensätze archiviert werden und insbesondere für die Auswertungen verfügbar bleiben.

Die Benutzerverwaltung erfolgt im SP auf Basis des Bündnis-Forest (BF; Active-Directory-Verbund staatlicher Dienststellen in Bayern), welches den Rückgriff auf eine Vielzahl der vorhandenen Benutzergrundeinstellungen ermöglicht. Die GÄ sind nicht im BF angelegt und erhielten als Teil des Behördennetzes einen eigenen Zugang durch das IT-DLZ, um so ins BF integriert zu werden und die Vorzüge des BF nutzen zu können.

Die GÄ legitimieren die Leistungserbringer durch die Anlage eines Untersuchungsdatensatzes auf dem SP (un)mittelbar zur Durchführung der Corona-Tests und damit auch zur Eingabe der Testergebnisse. In diesem Datensatz legen die GÄ auf Basis der rechtlichen Vorgaben (TestV) die Einrichtung fest, in der getestet wird, sowie den Leistungserbringer, den Testzeitraum, wann die (ersten) Tests stattfinden und bei Bedarf eine Meldehäufigkeit und -frequenz (sog. Terminfolgen) für den automatischen Versand der Eingabelinks. Der SP erzeugt beim Speichern des Datensatzes automatisch ein „Secret“ und verschickt dieses per E‑Mail als Linkbestandteil an den hinterlegten Leistungserbringer. Das „Secret“ ist eine zufällig systemseitig generierte Zeichenkette (vergleichbar mit einem Einmal-Token) und dient der Absicherung der Datenübermittlung. Es können somit grundsätzlich keine Daten von unautorisierter Seite an die SP-Datenbank gesendet und auf eine aufwendige Authentifizierung (Benutzername und Passwort) verzichtet werden. Der Leistungserbringer erhält zum Meldezeitpunkt den o. g. Link zur Dateneingabe im Eingabeformular.

### Formularserver

Wird der in SP generierte und per E‑Mail verschickte Link ausgewählt, gelangt der Leistungserbringer ohne Zugang zum Behördennetz oder weitere Berechtigungsnachweise zu seinem FS-Eingabeformular für die Testergebnisse. Die Datenübertragung erfolgt https-verschlüsselt über das Internet. Die Daten werden nur dann vom FS in den SP-Datensatz übernommen, wenn das „Secret“ dort existiert und bisher nicht übermittelt wurde. Die aggregierten und anonymisierten Testergebnisse ohne Personenbezug werden nach Eingabe durch den Leistungserbringer in BayCoRei übertragen und dem entsprechenden Untersuchungsdatensatz zugeordnet.

### Oracle-Datenbank

Aufgrund der gestiegenen Datenmenge werden die im SP eingetragenen Daten zur Sicherstellung der SP-Performance tagesaktuell per Schnittstelle automatisiert in die OD exportiert und monatlich im SP gelöscht. Die Daten der OD werden mittels standardisierter Exporte für Datenanalysen oder Reportings zur Verfügung gestellt und mit weiteren Auswertungsprogrammen (z. B. RStudio) weiterverarbeitet.

## Einführung des Meldeportals (Rollout)

Wenige Tage nach der Beauftragung wurde die Entwicklung der BayCoRei-Kernanwendung durch das IT-DLZ abgeschlossen und eine 3‑Systemlandschaft (Entwicklungssystem, Testsystem und Produktivsystem) bereitgestellt. Die vereinbarten Änderungen (Updates/-grades) werden nach der Programmierung im Entwicklungssystem durch das IT-DLZ ins Testsystem eingespielt und durch das LGL auf Funktionalität überprüft. Nach etwaiger Fehlerkorrektur erfolgen die LGL-Abnahme und die anschließende Übernahme in das Produktivsystem. Im Testzyklus können Fehler vor einer Einführung in den Realbetrieb korrigiert sowie dynamisch Anpassungen und Verbesserungen vorgenommen werden. So wurden bspw. in der initialen Testphase Einrichtungslisten (Krankenhäuser, Heime u. a.) vom LGL aufbereitet und per Skript zur Entlastung der GÄ vom IT-DLZ eingespielt und die von Beginn an angedachten Terminfolgen zügig umgesetzt. Zur Vorbereitung des Rollouts erfolgte ein Probelauf mit einem GA, um auftretende Probleme aus Sicht der GÄ abschätzen zu können und weitere Verbesserungen der Benutzerfreundlichkeit vorzunehmen.

Zum Zeitpunkt des Rollouts wurden Unterstützungsmaterialien (z. B. Erklärvideos und Schnellstarthilfen) zur Handhabung des Meldeportals bereitgestellt, stetig aktualisiert und verbessert. Der am LGL eingerichtete First-Level-Support (E-Mail und Telefon) ist wie üblich erster Ansprechpartner und beantwortet auftretende Fragen der Leistungserbringer und GÄ. Mit den unterschiedlichen Unterstützungshilfen sollte die schnelle Einführung einer neuen digitalen Lösung in der pandemiebedingt angespannten Situation erleichtert und Nutzergruppen mit teilweise geringen technischen Vorkenntnissen unterstützt werden.

## Betrieb und Weiterentwicklung von BayCoRei

Im laufenden Betrieb hat sich die gewählte Technik, auch hinsichtlich der mannigfaltigen Anpassungsbedarfe bewährt. Erfolgsentscheidend war dabei auch der regelmäßige fachliche Austausch zu den rechtlich-, technisch-, benutzerfreundlichkeits- und sicherheitsbedingten Anpassungen des Meldeprozesses zwischen dem LGL und dem IT-DLZ sowie dessen langfristige und priorisierte Unterstützung.

**Figure 4:**
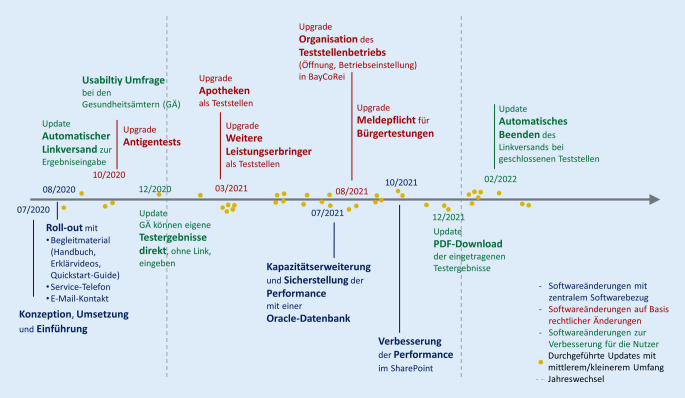

Abb. 4 zeigt sowohl die größeren Anpassungsbedarfe von BayCoRei als auch die vielen arbeitsintensiven, wenn auch kleineren Updates. Die Grafik soll einen Eindruck von der Häufigkeit der geforderten Anpassungen und der damit verbundenen notwendigen Agilität der Lösung vermitteln. Aufgrund der pandemiebedingt galoppierenden Einführung von BayCoRei wurden Anpassungen bereits während der Einführung vorbereitet und permanent über die gesamte Laufzeit hinweg zur Sicherstellung der Performance erörtert. Zur Bewältigung der Vielzahl und der dichten Taktung der Updates/Upgrades wurden all diese auch während des Betriebs zwingend nach einem standardisierten Prozess getestet. Die Umsetzung erfolgte qualitätsgesichert und wurde über diverse Informationskanäle und mit verschiedenen Materialien begleitet.

Die größten Änderungen waren die TestV-bedingte Aufnahme der AG-Tests, die Aufnahme weiterer Leistungserbringer und die Meldepflicht für alle Leistungserbringer von Bürgertestungen nach der TestV. Aber auch kleinere fortwährende Änderungen bzgl. der aktuellen rechtlichen Anforderungen anzupassenden Kategorien, Berichtsfrequenzen, Obergrenzen der Testanzahl sowie die einzugebenden Pflichtfelder waren beträchtlich. All diese Weiterentwicklungen bedürfen für eine erfolgreiche Umsetzung letztendlich der fachlichen Absprache, ausführlicher Informationen für die GÄ und Leistungserbringer und deren Unterstützung bei den Eintragungen, aber auch der Akzeptanz der Nutzer.

## Steuerung der Pandemiemaßnahmen mithilfe vom BayCoRei

Neuinfektionen werden als Faktor zur Steuerung der Pandemiemaßnahmen herangezogen. Eine erweiterte Teststrategie könnte sodann einen weiteren wichtigen Baustein in der Pandemiebekämpfung darstellen. Verbunden mit einem bevölkerungsbezogenen Monitoring an eine zentrale Stelle könnten diese Daten genutzt werden, um das aktuelle Pandemiegeschehen besser einschätzen und schnell reagieren zu können [5]. Wie bereits beschrieben, stand bei BayCoRei die technische Lösung zur systematischen Erfassung der Testergebnisse im Vordergrund. Neben der Technik könnten vor allem die Verbindlichkeiten sowie die Akzeptanz und Umsetzung durch die Akteure einen Einfluss darauf haben, ob die eingegangenen Daten vollständig und valide erfasst und als politische Entscheidungshilfe herangezogen werden können.

Seit dem 01.08.2021 sind laut TestV lediglich die Ergebnisse der Bürgertests meldepflichtig in BayCoRei einzutragen. Andere Testergebnisse von Reihenuntersuchungen für vulnerable Personengruppen nach der TestV sind bis heute nur berichtspflichtig. Dennoch ist die Meldequote bei den Leistungserbringern für Bürgertests vergleichbar oder sogar niedriger (z. B. Apotheken im Durchschnitt 62 %, weitere Leistungserbringer 70 %, Arztpraxen 43 %) als die der „nur“ berichtspflichtigen Krankenhäuser (77 %) oder Rehabilitationseinrichtungen (78 %). Selbst die notwendige Registrierung und Meldung der Testergebnisse nach der TestV, um sich an die CWA anbinden und mit der KVB abrechnen zu können, haben die Meldequote nicht sichtbar beeinflusst.

Aufgrund der wöchentlichen bzw. monatlichen Melde‑/Berichtsfrequenz oder auch der ausbleibenden Übermittlung der Testuntersuchungen kann BayCoRei nicht zur Echtzeitsteuerung der Pandemie herangezogen werden. Das Fehlen einzelner Tests oder ganzer Testuntersuchungen sowie die Qualität der Eingaben lassen sich nur schwer valide abschätzen. Dennoch kann BayCoRei mittels regelmäßiger Berichte und Auswertungen, mit höchster Vorsicht bei der Dateninterpretation, ein weiteres Informationsmerkmal und eine Orientierungshilfe für politische Entscheidungsträger sein.

Im Reporting an die GÄ, Regierungen und Vertreter des Ministeriums werden seit Oktober 2020 wöchentlich bzw. monatlich für alle Einrichtungen die in BayCoRei erfasste Gesamtzahl der AG- und PCR-Tests und die Positivquoten nach Anlass und Art der Einrichtung sowie nach Region dargestellt. Die Abbildungen können genutzt werden, um regionale Muster und zeitliche Verläufe von Neuinfektionen zu erkennen und ggf. im Kanon mit anderen Indikatoren Maßnahmen zu ergreifen.

## Diskussion

Wie im Abschnitt Bayerisches Meldeportal für Corona-Reihenuntersuchungen erläutert, wurde mit BayCoRei eine einfache Lösung zur Datenerfassung geschaffen, die bzgl. ihrer Grundfunktionalitäten technisch schnell aufgesetzt und sehr dynamisch weiterentwickelt werden kann sowie den ständig wechselnden Gegebenheiten einer Pandemie gemeinhin standhalten soll. Durch die Verfügbarkeit eines einheitlichen Systems waren Insellösungen für einzelne GÄ entbehrlich und Daten für die Abrechnungsprüfung durch die KVB zentral grundsätzlich verfügbar.

Auch wenn der ExpertenInnenrat der Bundesregierung zu COVID-19 in der 11. Stellungnahme zur Steuerung und Bewältigung der Pandemie einen wesentlich umfassenderen Werkzeugkasten und andere, weiterreichende Steuerindikatoren empfiehlt [6], könnte die vorliegende Lösung auch als einzelnes Steuerungswerkzeug – ggf. im Verbund mit anderen Erfassungssystemen – das Management der Pandemie unterstützen, wenn die Daten vollständig, valide und in Echtzeit erfasst würden. In der aktuellen Form sind, wenn überhaupt, nur retrospektive Analysen mit der gebotenen Vorsicht bei der Dateninterpretation möglich, weil die Daten unvollständig und zeitversetzt erfasst werden (Selektions- und Informationsbias, vergleiche bspw. [7]).

Gleichzeitig schließt ein erkannter systematischer Fehler bei verständiger Interpretation einen Nutzen durch die Analyse auch unvollkommen erhobener Daten nicht aus. Eine umfassende Meldepflicht für alle Leistungserbringer könnte u. U. zu einem vollständigeren Datensatz beitragen. Auch bei den etablierten Erfassungssystemen für die gesetzlich meldepflichtigen Infektionskrankheiten ist von Untererfassungen auszugehen. Vergleichsuntersuchungen nach der „Rückfangmethode“ (Capture-Recapture Method) zeigten, dass bei der invasiven Meningokokkenerkrankung 90 % der tatsächlich ärztlich diagnostizierten Fälle, bei der Creutzfeldt-Jakob-Krankheit lediglich 60 % und bei Echinokokkosen nur 30 % der Fälle gemeldet werden [8–10]. In Kenntnis bzw. bei ausreichend valider und präziser Abschätzung der möglichen Erhebungsfehler (Ascertainment-Bias) sind demnach Aussagen zum zeitlichen Verlauf und ggf. auch zu regionalen Variationen dennoch möglich.

Die Untererfassung in BayCoRei könnte durch die hohe Belastung der GÄ und Leistungserbringer erklärt werden. Die GÄ können die einmalige Anlage der Einrichtungen/Leistungserbringer nicht (immer) zeitnah vornehmen. Die Leistungserbringer können die Testergebnisse folglich nicht eingeben bzw. melden sie aufgrund von eigenen Überlastungen, trotz vorheriger Anlage durch das GA, nicht (vollständig) an BayCoRei. Zudem wird die Nichterfüllung der Meldepflicht nicht sanktioniert. In einigen Fällen könnte auch die mangelnde Kenntnis des erforderlichen Meldewegs zum Versäumen der Meldepflicht führen.

Zu fragen ist nun, ob es Handlungsansätze gibt, um BayCoRei als digitales Instrument zur Unterstützung eines umfassenderen Steuerungswerkzeugs auszugestalten und inwieweit hier eine Orientierung an Benchmarks möglich ist. Dazu können naheliegenderweise andere Online-Portale, die bereits in die Steuerung von Pandemiemaßnahmen eingebunden sind, herangezogen werden. 2 Beispiele sind in Tab. 2 näher beschrieben: das Bayerische Digitale Pflege-Portal (BayDiPP) und der Interdisziplinäre Versorgungsnachweis (IVENA). Beide Portale greifen auf bereits etablierte Systeme zurück und wurden entsprechend der Zielsetzung des jeweiligen Portals weiterentwickelt. Diese Meldeportale sind an eine verpflichtende Meldung gekoppelt. Aufgrund entsprechender finanzieller Unterstützung bei der Versorgung von COVID-19-Patienten (Versorgungsaufschlag) kann von einer hohen Meldemotivation der auf SARS-CoV‑2 positiv getesteten Patienten in IVENA ausgegangen werden, wohingegen BayCoRei und BayDiPP die Nutzer lediglich durch die rechtlichen Rahmenbedingungen zum Erfüllen der Meldepflicht motivieren können.

**Table 2:** 

	Bayerisches Digitales Pflege-Portal (BayDiPP)	Interdisziplinärer Versorgungsnachweis (IVENA)
Zielsetzung	Verpflichtende tagesaktuelle, digitale Erfassung von** Ausbruchsgeschehen** (Anzahl und Intensität der Ausbrüche) in **bayerischen Pflegeeinrichtungen**	Verpflichtende tagesaktuelle Meldung von **Behandlungs- und Bettenkapazitäten** der bayerischen **Krankenhäuser**
Einbindung in die Steuerung der Pandemiemaßnahmen	Einführung des technischen Tools am 19.01.2021	Seit dem Inkrafttreten der Allgemeinverfügung zur Bewältigung erheblicher Patientenzahlen in Krankenhäusern am 25.03.2020 [11]
Erfasste Merkmale	Angaben und Fallzahlen zu Ausbruchsgeschehen (z. B. Gesamtanzahl COVID-19-positive Fälle, hospitalisierte SARS-CoV-2-positive Fälle, Todesfälle an und mit COVID-19, Testungen)	Differenzierung nach Intensiv‑, Intermediate-Care- und Normalpflegestation, für jedes Krankenhaus: Aktuell betriebene Betten Maximale Bettenkapazitäten Belegte und freie Betten, mit und ohne Patienten mit (Verdacht auf) SARS-CoV‑2 Belegte und freie Betten mit ECMO (extrakorporale Membranoxygenierung; Lungenunterstützung außerh. des Körpers) SARS-CoV-2-Erstaufnahmen (jew. bezogen auf Erwachsene)
	Angaben zur aktuellen Situation in der pflegerischen Versorgung	
Komponenten/Architektur	Schnelle Entwicklung unter Verwendung des bereits vorhandenen Tools (Formularserver) in Kombination mit Eigenentwicklungen: Webanwendung zur Datenerfassung und Datenübertragung Nutzeroberfläche (Portal) für Bearbeiter, um die aktuelle Situation, Trends und neue Ausbrüche zu erkennen Vorhandene Datenbank im Hintergrund	Entwickelt von der mainis IT-Service GmbH: Webanwendung/internetbasiertes System Hochverfügbarkeit und Performance Erhöhte Ausfallsicherheit Software-Architektur, Programmierung und Infrastruktur sind auf anwendungsgerechte Antwortzeiten und Verfügbarkeiten konzipiert
(Weiter‑) Entwicklung	Seit 2020 regelmäßige Anpassung der Eingabemaske und der Datenbank an neue Anforderungen und Erkenntnisse	Entwicklung des IVENA-Sonderlage-Moduls zur Unterstützung bei der Bewältigung der Corona-Pandemie
	Etablierung als Frühwarnsystem geplant	Gelegentliche Anpassung der zu erfassenden Daten
Beitrag zur Steuerung von Pandemiemaßnahmen	Trendaussagen (z. B. Anzahl der Todesfälle, hospitalisierte Fälle der letzten 7 Tage), grafische Aufbereitung	Regelmäßiges Berichtswesen zu zahlreichen Parametern
	Regelmäßiges aktuelles Monitoring von Ausbruchsgeschehen für die fortwährende Neubewertung von Schutz- und Hygienemaßnahmen in den Pflegeeinrichtungen	Spezialauswertungen für die Politik
	Langfristige Weiterentwicklung zu einem Frühwarnsystem	Landesweite Belegungssteuerung im Rahmen der systematischen Koordinierungsaktivitäten
	Datenquantität: Tägliche Aktualisierung durch die Kopplung an den Katastrophenfall gewährleistet	Laufendes Monitoring des Infektionsgeschehens zur Unterstützung der ressourcenoptimalen Verteilung der Patienten und zur Ergreifung zusätzlicher Maßnahmen
	Datenqualität: u. a. durch das LGL überwacht, Meldefehler werden korrigiert, es besteht u. a. kein Eingabeschutz für unplausible Werte, wie z. B. Tippfehler	
		Datenquantität: Tägliche Aktualisierung auf Basis der Allgemeinverfügung verpflichtend
		Datenqualität: u. a. durch das LGL überwacht; es besteht u. a. kein Eingabeschutz für unplausible Werte, wie z. B. Tippfehler; verspätete bzw. versäumte Meldungen sind identifizierbar

*LGL* Bayerisches Landesamt für Gesundheit und Lebensmittelsicherheit

Vor dem dargelegten Hintergrund wären bei der Entwicklung bzw. beim Einsatz einer Meldesoftware neben den Überlegungen zur technischen Ausgestaltung und der Unterstützung der Nutzer insbesondere die Meldevoraussetzungen und die Thematik der Meldepflicht zu beleuchten sowie Anreize für deren Einhaltung zu setzen. Einerseits scheinen verpflichtende Meldungen vor allem die Vollständigkeit der Daten (Datenquantität) sicherzustellen, wie die gerade gezeigten Beispiele verdeutlichen, anderseits wird auch bei einer gesetzlichen Meldepflicht, wie weiter oben erläutert, keine Meldequote von 100 % erreicht. Des Weiteren ist neben der Datenquantität (Responserate) auch bei vorhandenen grundlegenden Qualitätssicherungsinstrumenten die Datenqualität fortlaufend aufmerksam zu (über-)prüfen.

## Fazit

Der Katalysator SARS-CoV-2-Pandemie hat gezeigt, dass eine digitale Lösung in einem komplexen Umfeld schnell umsetzbar sein kann. Voraussetzungen dafür sind, dass die Notwendigkeit einer schnellen technischen Lösung besteht, Ressourcen für die Konzeption, Umsetzung und Weiterentwicklung priorisiert werden, die Beteiligten an einem Strang ziehen und mit einem minimalen Funktionsumfang gestartet werden kann, der dann iterativ weiterentwickelt wird. Zur (Echtzeit‑)Steuerung der Pandemie sind allerdings weitere ineinandergreifende kritische Faktoren von zentraler Bedeutung, wie z. B. verbindliche Meldepflichten, ausreichende Personalressourcen der Datenerfasser, Vermeidung von teilredundanter Datenerfassung in unterschiedlichen Lösungen, Qualitätssicherung und Nutzerbetreuung. Sie sind bei jeder Maßnahme eigens auszuloten.

Unbenommen dessen, dass weitergreifende konzeptionelle Arbeitsgruppen andere Steuerungsindikatoren zur Etablierung vorgeschlagen haben bzw. etablieren werden (z. B. Abwassermonitoring), können die Grundidee und die Vorgehensweise von BayCoRei beispielhaft für die Konzeption, Entwicklung und Umsetzung digitaler Lösungen herangezogen werden. BayCoRei ist eine schnell umsetzbare und hochagile digitale Lösung, die eine einheitliche Datenerfassung ermöglicht.

## Footnotes

1 „Gesetz zur Verhütung und Bekämpfung von Infektionskrankheiten beim Menschen“.

2 Ab 15.10.2020, zuvor „Verordnung zum Anspruch auf bestimmte Testungen für den Nachweis des Vorliegens einer Infektion mit dem Coronavirus SARS-CoV-2“ (Rechtsverordnung vom 08.06.2020).

3 Software, die mit einem möglichst minimalen Funktionsumfang veröffentlicht wird, damit das Produkt schnell zur Verfügung steht. Die weitere Entwicklung erfolgt während der Nutzung stufenweise und bedarfsorientiert gemeinsam mit dem Nutzer [3].
